# Effect of V_2_O_5_ B-site substitution on the microstructure, Raman spectrum, and dielectric properties of SrBi_2_Ta_2_O_9_ ceramics

**DOI:** 10.1038/s41598-020-73327-2

**Published:** 2020-11-05

**Authors:** Chia-Ching Wu, Cheng-Fu Yang

**Affiliations:** 1 Department of Applied Science, National Taitung University, Taitung, Taiwan, R.O.C.; 2 Department of Chemical and Materials Engineering, National University of Kaohsiung, Kaohsiung, Taiwan, R.O.C.

**Keywords:** Electrical and electronic engineering, Engineering, Materials science

## Abstract

Strontium bismuth tantalate vanadate [SrBi_2_(Ta_2−x_V_x_)O_9_, SBTV] ceramics, which are bismuth-layered perovskite ferroelectrics, were synthesized through the solid-state reaction method. The effects of different sintering temperatures and V_2_O_5_ contents on the structure of the microstructure, Raman spectrum, and dielectric properties of the SBTV ceramics were investigated. As sintered at high temperature (980–1040 °C) and different V_2_O_5_ contents (x = 0.1 − x = 0.4), only disk-like grains of the SBTV ceramics were observed in the scanning electron micrographs. Preferential orientation of the crystals of the SBTV ceramics was confirmed through X-ray diffraction studies. The higher dielectric constant and Curie temperature of the SBTV ceramics compared with those of strontium bismuth tantalite (SrBi_2_Ta_2_O_9_, SBT) ceramics are ascribe to the partial replace of Ta^5+^ ions by V^5+^ ions in the B sites. The Curie–Weiss law and the modified Curie–Weiss law were used to discuss the normal-type or relaxor-type ferroelectric characteristic of the SBTV ceramics. The Ta^5+^ ion replaced by V^5+^ ion site in SBT ceramics to form SBTV ceramics exerted a pronounced effect on the BO_6_ mode, as demonstrated by Raman spectrum results.

## Introduction

Ferroelectric materials, a technologically important group of materials, exhibit various favorable phenomena. For example, these materials have high permittivity, high piezoelectric and pyroelectric coefficients, optimal electro-optic properties, and reliable polarization switching^1^. Ferroelectric materials are used in a wide range of applications and devices, such as in infrared detectors, nonvolatile ferroelectric memory, dynamic random access memory, decoupling capacitors, and high Q resonators^2–5^. Bismuth layer-structured ferroelectric (BLSF) compounds are a crucial group of ferroelectric materials. The layer-type bismuth ferroelectric group was first studied by Autivillius in 1949^6^, who synthesized numerous compounds such as Bi_3_Ti_4_O_12_, BaBi_2_Ta_2_O_9_, and CaBi_2_Ta_2_O_9_. Layered perovskite strontium bismuth tantalite (SrBi_2_Ta_2_O_9_, SBT), an alternative compound in the layer-type bismuth family, has been attracting considerable scientific and commercial attention due to its excellent properties that facilitate their use in dynamic random access memory^7–9^. SBT ceramics belong to the Aurivillius family of bismuth-layered perovskites, with their general formula being (Bi_2_O_2_)^2+^(A_m−1_B_m_O_3m+1_)^2−^, where A = Na^+^, K^+^, Ca^2+^, Sr^2+^, Ba^2+^, Bi^3+^, etc.; B = Ti^4+^, Nb^5+^, Ta^5+^, Mo^6+^, W^6^^+^, Fe^3+^, etc.; and m = 1–5^10,11^. However, the major disadvantages of SBT ceramics are that they have low remanent polarization and require high processing temperatures^12^. Researchers have enhanced ferroelectric properties of SBT by using SBT-(Bi_4_Ti_3_)_1−*x*_Nb_*x*_O_12_ (SBT-BTN) multilayer thin films^13^. Wu et al.^14^ reported a reduction in the necessary processing temperature by conducting partial substitution of Nb^5+^ ions with V^5+^ ions in SrBi_2_Nb_2_O_9_ ceramics. SrBi_2_(Nb,V )_2_O_9_ ceramics crystallize at a low temperature and have enhanced dielectric properties. Furthermore, in strontium bismuth niobite ferroelectrics, the partial substitution of strontium with other cations such as calcium and lanthanum improve their dielectric and electrical properties, for example, the para-ferroelectric transition temperature^15^.

Although SBT ceramics are slightly less polarized than are the competing lead zirconate titanate (PZT)-based materials, bismuth-layer compounds are more robust to polarization fatigue; that is, almost no charge loss occurs when polarization is reversed after many cycles. Lead (Pb) causes high environmental pollution. In February 2003, the European Union adopted the Restriction of Hazardous Substances Directive (RoHS) to regulate the use of certain hazardous substances in electrical and electronic equipment.

In this study, V_2_O_5_ was used as a substitute for Ta_2_O_5_ in SBT ceramics to form SrBi_2_(Ta_2−x_V_x_)O_9_ ceramics. Through partial substitution of Ta^5+^ ions with V^5+^ ions, the sintering temperature was reduced and the ferroelectric properties of SBTV ceramics were enhanced. The effects of sintering temperature and V_2_O_5_ content on the microstructure, Raman spectrum, and dielectric properties of SrBi_2_(Ta_2−x_V_x_)O_9_ ceramics were investigated. Raman scattering a sensitive technique for investigating the lattice vibrational modes that can provide information for identifying changes in lattice vibrations and information about the positions occupied by the added ions was used to study the effect of adding V_2_O_5_ in SBT ceramics.

## Experimental procedures

SrBi_2_(Ta_2−x_V_x_)O_9_ (SBTV) ceramics were synthesized using a solid-state reaction method based on the following chemical reaction:

1 $${\text{SrCO}}_{{3}} + {\text{ Bi}}_{{2}} {\text{O}}_{{3}} + {\text{ Ta}}_{{2}} {\text{O}}_{{5}} \to {\text{SrBi}}_{{2}} {\text{Ta}}_{{2}} {\text{O}}_{{9}} + {\text{ CO}}_{{2}} \uparrow$$

When Ta_2_O_5_ is substituted by V_2_O_5_, and the equation can be modified as follow:

2 $${\text{SrCO}}_{3} + {\text{ Bi}}_{2} {\text{O}}_{3} + {(1} - {\text{x) Ta}}_{2} {\text{O}}_{5} + {\text{ x V}}_{2} {\text{O}}_{5} \to {\text{SrBi}}_{2} \left( {{\text{Ta}}_{{{2} - {\text{x}}}} {\text{V}}_{{\text{x}}} } \right){\text{O}}_{9} + {\text{ CO}}_{2} \uparrow$$

Reagent-grade raw materials SrCO_3_, Bi_2_O_3_, Ta_2_O_5_, and V_2_O_5_ with purity higher than 99.5% were used as precursors. They were mixed per the composition of the SBTV (x = 0.1 to x = 0.4) ceramics and ball-milled for 2 h with deionized water. After being dried and ground, the powder was calcined at 850 °C for 4 h. After calcination, the powder was ground again. The obtained powder was uniaxially pressed into pellets in a steel die that had a thickness of 1 mm and a diameter of 12 mm. These pellets were sintered at 920–1040 °C for 8 h. The microstructure of the SBTV ceramics were observed through scanning electron microscopy (SEM). The crystalline structure of the SBTV ceramics were investigated through X-ray diffraction (XRD) studies using CuKα radiation. SBTV ceramics were analyzed through Raman spectroscopy using the 532-nm line of a YAG laser operated at a power of 100 mW from 100 to 1100 cm^−1^. The laser had a beam diameter of approximately 1.8 μm and was focused on the SBTV ceramics surface using a 1000 × objective lens. Before the dielectric properties of the SBTV ceramics were characterized, its surface was painted with Ag–Pd paste and sintered at 600 °C by using as electrodes. Temperature-dependent dielectric characteristics of the SBTV ceramics were measured at a frequency of 1 MHz by using the HP4294 impedance analyzer in a temperature-programmable testing chamber.

## Results and discussion

**Figure 1:**
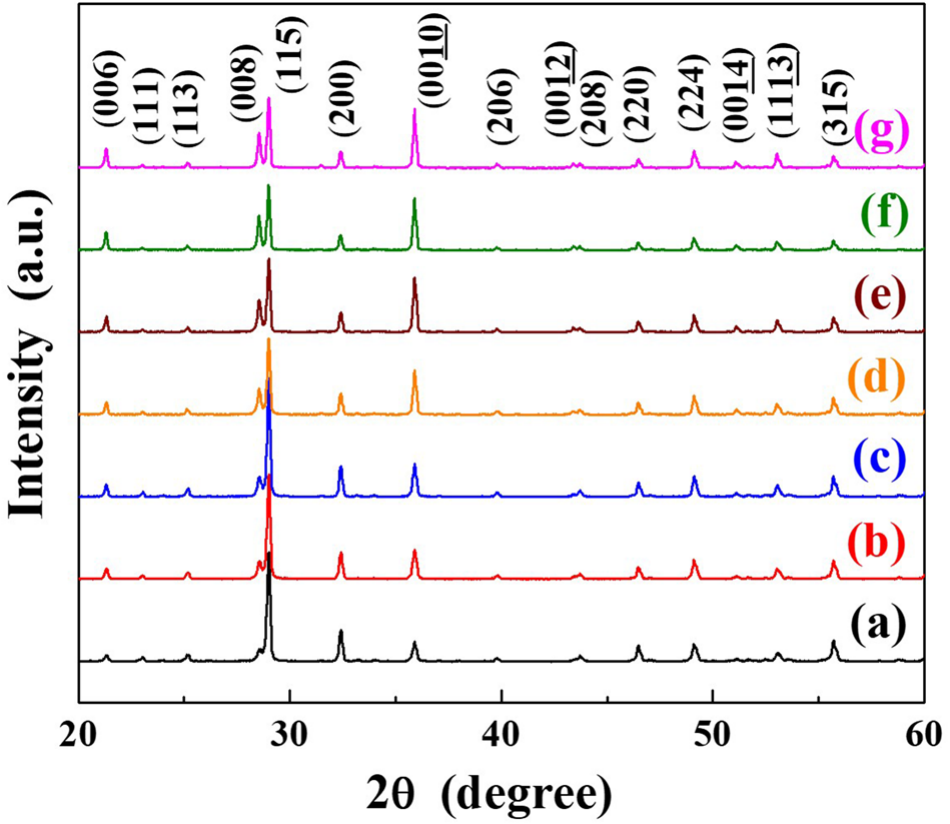
X-ray diffraction patterns of SrBi_2_Ta_1.9_V_0.1_O_9_ ceramics sintered at different temperatures. (**a**) 920 °C, (**b**) 940 °C, (**c**) 960 °C, (**d**) 980 °C, (**e**) 1000 °C, (**f**) 1020 °C, and (**g**) 1040 °C, respectively.

Phase identification of the SrBi_2_(Ta_2−x_V_x_)O_9_ (x = 0.1, abbreviation SBTV1) ceramics sintered at various temperatures were conducted by X-ray diffraction (XRD) analysis, as shown in Fig. 1. The diffraction peaks of the SBTV1 ceramics were similar with the standard data of the SBT (JCPDS no. 49-0609) ceramics. In addition, no any secondary or unknown phases were observable in the SBTV1 ceramics as sintered at various temperatures, only the single-phase layered perovskite structure was present. These results suggesting that the Ta^5+^ ions were substituted by V^5+^ ions in the SBTV1 ceramics. In the (00*l*) preferred orientations, the 2θ = 21.39°, 28.63°, 35.91°, 43.42°, and 51.08° can be attributed to the (006), (008), (0010), (0012), and (0014) planes of the SBTV1 ceramic, respectively. The diffraction intensities of the (00*l*) planes increased and that of the (115) plane decreased as the sintering temperature was increased (Fig. 1), particularly of the (008) and (0010) planes. Huanga et al. indicated that the diffraction intensities corresponding to (00*l*) preferred orientations (i.e.) (006), (008), (0010), (0012), and (0014) of the SBTV1 were stronger than those of undoped SBN ceramics^16^. V_2_O_5_ has a low melting point of 690 °C; thus, the results indicate that V_2_O_5_ has a liquid-phase effect during sintering to promote the crystal growth of the (00*l*) preferential orientation of the SBTV1 ceramics^17^. The degree of the (00*l*) preferential orientation can be calculated using the Lotgering method^18^:

3 $$P = \frac{{\sum I_{{\left\{ {00l} \right\}}} }}{{\sum I_{{\left\{ {hkl} \right\}}} }}$$

4 $$P_{0} = \frac{{\sum I_{{0\left\{ {00l} \right\}}} }}{{\sum I_{{0\left\{ {hkl} \right\}}} }}$$

5 $$F = \frac{{P - P_{0} }}{{1 - P_{0} }}$$

where *ΣI*_{*hkl}*_ and *ΣI*_{00*l}*_ are the summation of the diffraction intensities of the (*hkl*) and (00*l*) planes of the SBTV1 ceramics sintered at different temperatures, respectively. *P* can be measured using *ΣI*_{*hkl}*_ and *ΣI*_{00*l}*_ presented in Eq. (3). *ΣI*_*o*{*hkl}*_ and *ΣI*_*o*{00*l}*_ are the summation of the diffraction intensities of the (*hkl*) and (00*l*) planes of the SBT ceramics obtained from JCPDS. *P*_0_ can be measured using *ΣI*_*o*{*hkl}*_ and *ΣI*_*o*{00*l}*_ presented in Eq. (4). Here, *h*, *k*, and *l* are the Miller indices. The F factor is any value between 0 and 1, with 0 representing random orientation and 1 representing preferential orientation.

**Figure 2:**
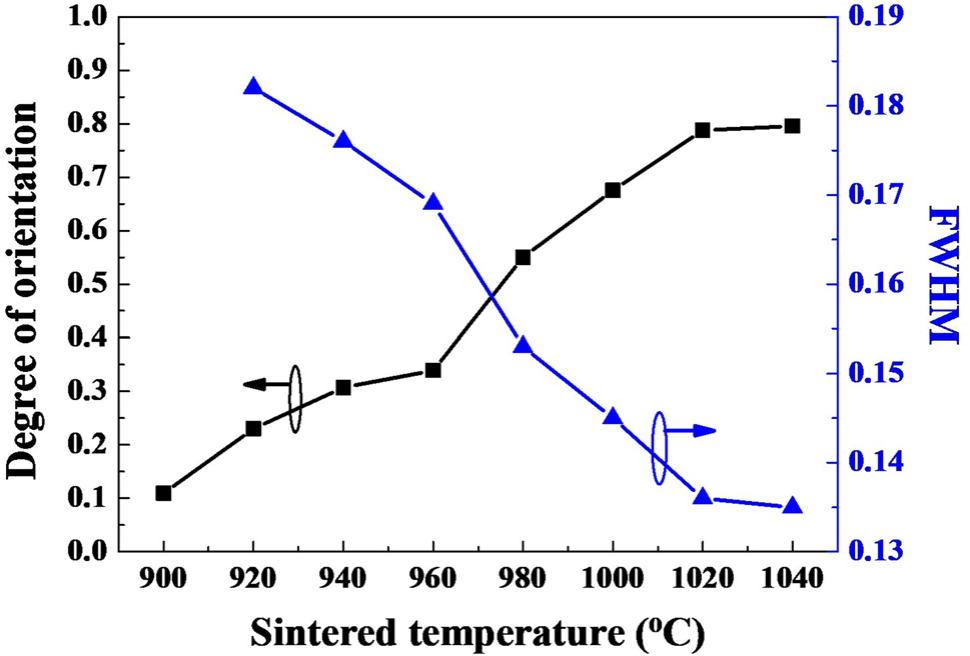
Degree of orientation and FWHM values of SrBi_2_Ta_1.9_V_0.1_O_9_ ceramics sintered at different temperatures.

Figure 2 shows the effect of the sintering temperature on the degree of preferential orientation (00*l*) peaks of the SBTV1 ceramics, calculated from the data in Fig. 1 by using Eqs. (3)–(5). The degree of the (00*l*) preferential orientation of the SBT ceramics was approximately 0.11 (Fig. 2), indicating that SBT ceramics are polycrystal in nature, with random orientation. Compared with SBT ceramics, the SBTV1 ceramics sintered at 920 °C had a higher degree of preferential orientation (i.e., 0.23) of the (00*l*) planes. The degree of orientation of the SBTV1 ceramics gradually increased with sintering temperature till 960 °C, beyond which it increased quickly. In this study, the maximum preferential orientation of 0.81 was obtained for SBTV1 ceramics sintered at 1040 °C. The major planes of the SBTV1 ceramics were parallel to the (00*l*) planes, which are deemed preferential growth in layered perovskites. As sintering temperature was increased from 920 to 1040 °C, the full width at half maximum (FWHM) for the peak of the (115) plane of the SBTV1 ceramics decreased from 0.182 to 0.135 (Fig. 2). These results suggest that as the sintering temperature increased from 920 to 1040 °C, the crystallization, preferential orientation, and grain size of the SBTV1 ceramics increased, and the variations in these parameters were in agreement with the variations observed in the SEM images presented in Fig. 6.

**Figure 3:**
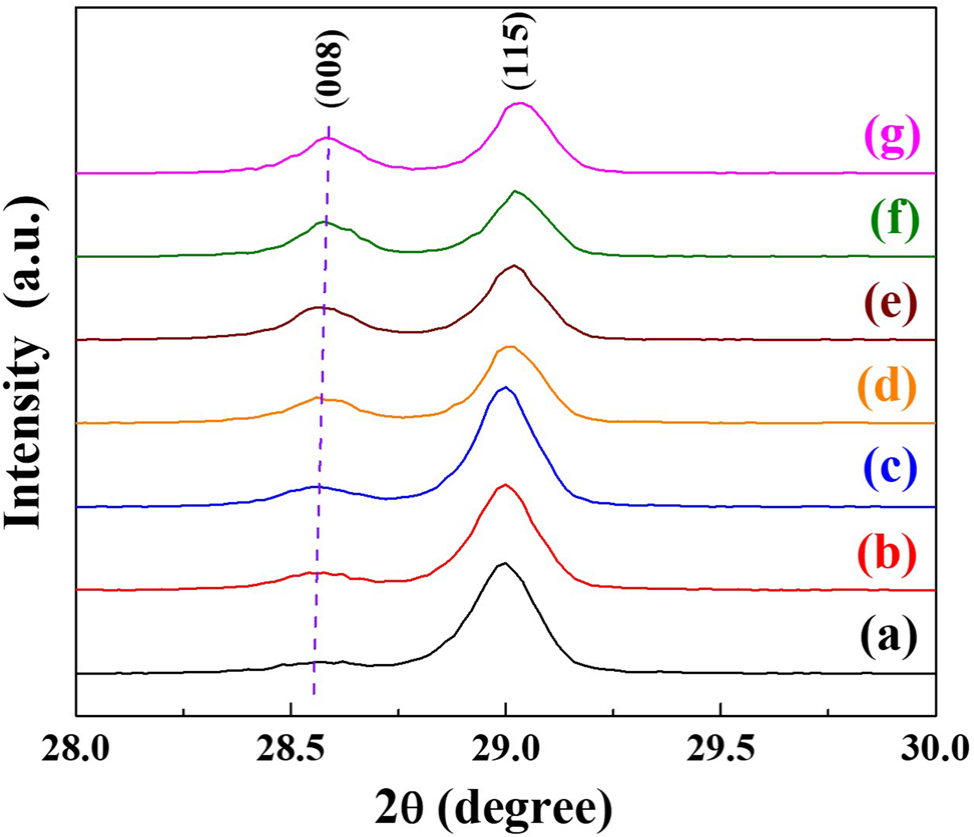
The grazing incidence angle X-ray diffraction patterns of the SBTV ceramics.

Figure 3 shows the grazing incidence angle X-ray diffraction patterns (GIAXRD) of the SBTV1 ceramics in the 2θ range of 28° to 30°. The diffraction peak of the (008) plane of the SBTV1 ceramics slightly increased from 2θ = 28.549° to 28.586° as the sintered temperature increased. According to Bragg’s law [nλ = 2d_(*hkl*)_sinθ], the d_(*hkl*)_ values slightly decreased from 3.131 Å to 3.124 Å with the increase in the sintered temperatures, indicating that the larger radius of Ta^5+^  = 0*.*64 Å can be substituted by smaller radius of V^5+^ = 0*.*54 Å, moreover, the number amount with Ta^5+^ ions were substituted by V^5+^ ions increased as the sintered temperature increased in the SBTV1 ceramics. Above results subsequently leads to the decreased lattice constant of the SBTV1 ceramics.

In order to further investigation of the *B*-site substitution of the SBTV1 ceramics, the crystal structure of SBTV1 ceramics was fitted using the SBT ceramic parameter (a = 5.5212 Å, b = 5.5215 Å, c = 24.992 Å). Rietveld refinement was carried out on the XRD data of the SBTV1 ceramics sintered at different temperatures, as shown in Fig. S1. The results show that the final refinement convergence was achieved with χ^2^ = 2.42, 3.41, 5.49, 6.67 as the sintering temperature at 920 °C, 960 °C, 1000 °C, and 1040 °C. From the Fig. S1, the different degree between the measurement and simulation results increased as the sintering temperature increases, especially in (006), (008), (0010) diffraction peaks. Crystal growth of the preferred orientation of (00*l*) planes was relationship to V_2_O_5_-doped in SBT ceramics.

**Figure 4:**
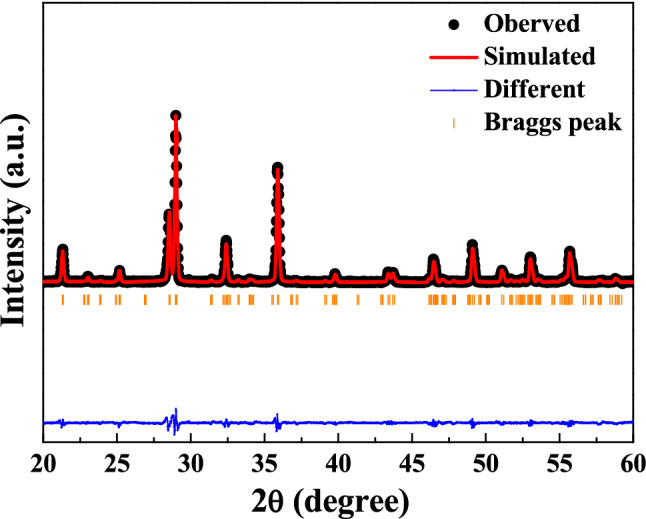
Rietveld refinement performed for the SrBi_2_Ta_1.9_V_0.1_O_9_ ceramic sintered at 1000 °C.

**Figure 5:**
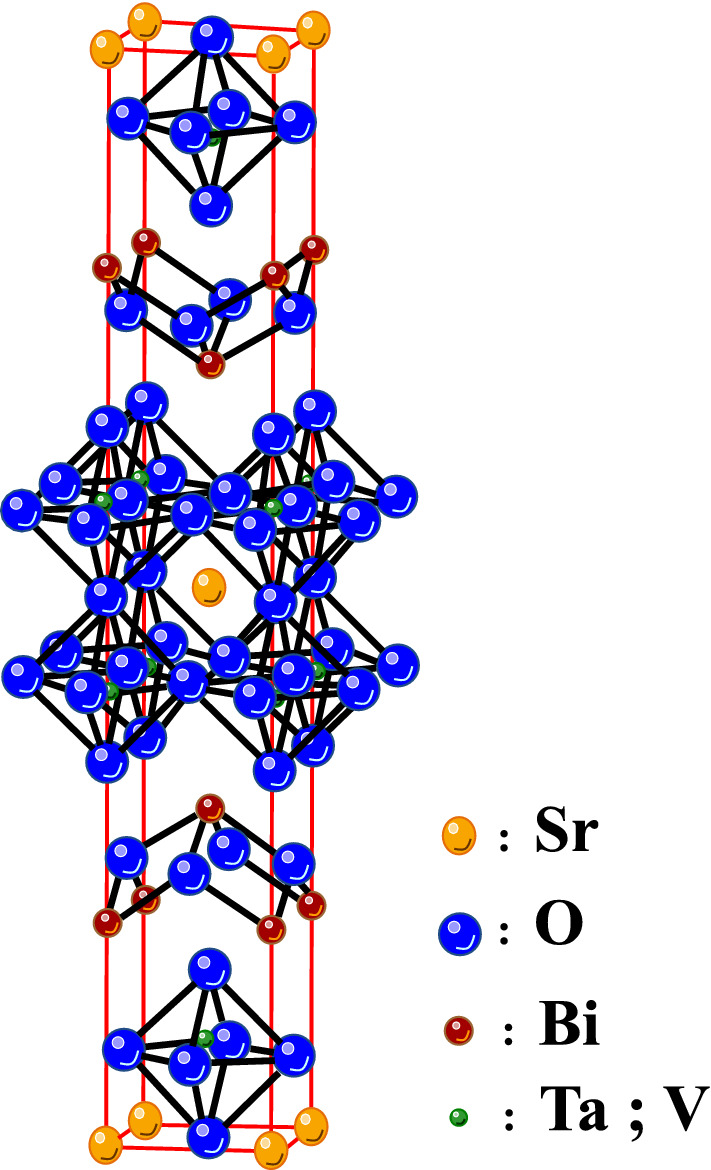
Atomic structure of the SrBi_2_Ta_1.9_V_0.1_O_9_ ceramic.

The crystal structure parameter of the SBTV1 ceramics sintered 1000 °C was fitted using the bismuth-layered perovskites structural model, the atomic positions being described in the space group A2_1_ *am*. The fitted profiles of the SBTV1 ceramics for XRD data sintered at 1000 °C is shown in Fig. 4. The final refinement convergence of the SBTV1 ceramics sintered 1000 °C was achieved with χ^2^ = 1.18 and the measured result was fitting to the simulation value. This result demonstrated that the Ta^5+^ ions were substituted by V^5+^ ions in the SBTV1 ceramics, as shown in Fig. 5.

**Figure 6:**
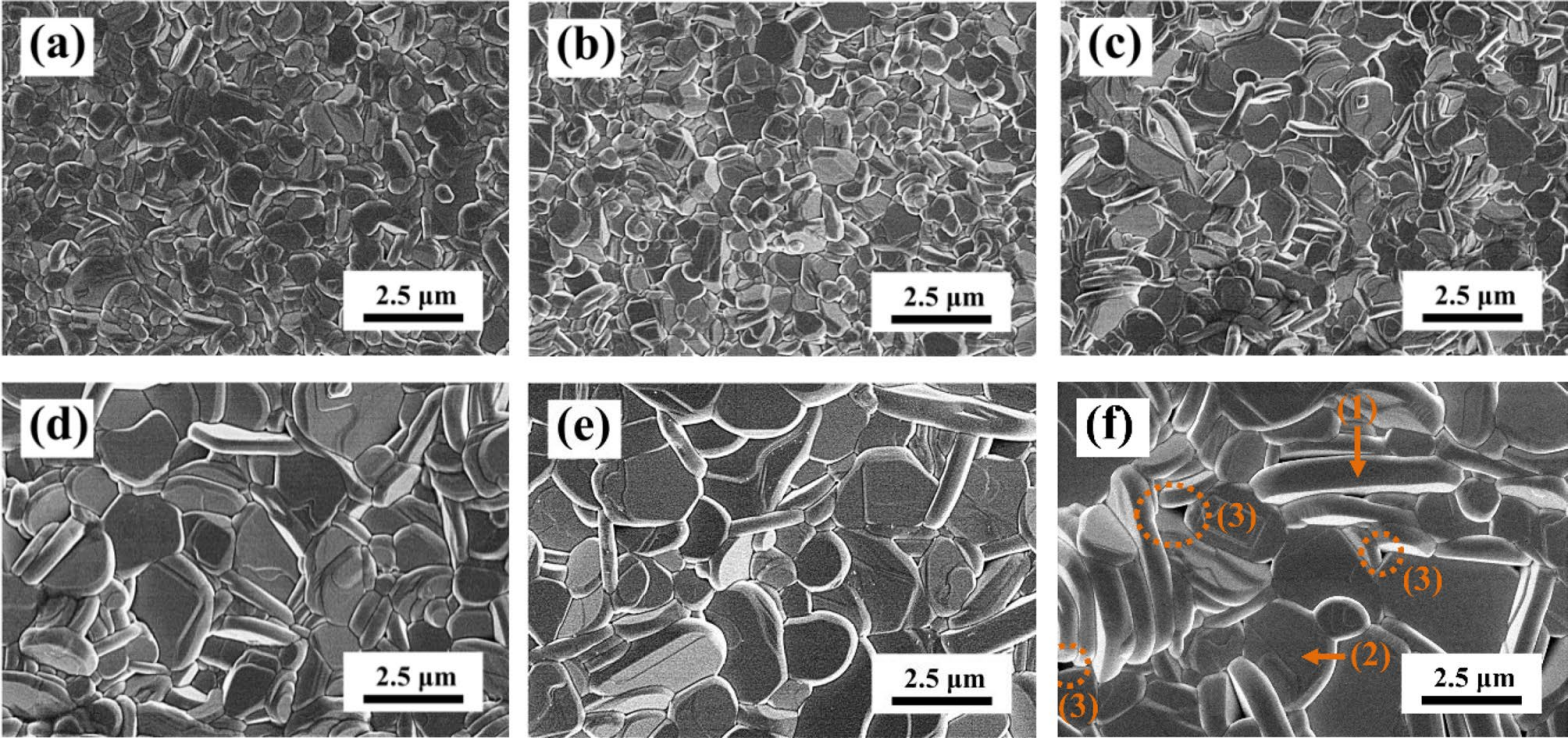
SEM images of SrBi_2_Ta_1.9_V_0.1_O_9_ ceramics under different sintering temperatures. (**a**) 940 °C, (**b**) 960 °C, (**c**) 980 °C, (**d**) 1000 °C, (**e**) 1020 °C, and (**f**) 1040°C, respectively.[Symbol: (1) Upright plate-like grain, (2) Horizontal plate-like grains and (3) Pore].

At a sintering temperature of 920 °C, the SBTV1 ceramic developed a porous structure, and grain growth was not observed (result not shown). The surface SEM morphologies of the SBTV1 ceramics were investigated as a function of sintering temperature (Fig. 6). When the sintered temperature below 980 °C, the morphologies of the SBTV1 ceramics has small grains, and grain growth was not very evident (Fig. 6a,b). The plate-like grains of the SBTV1 ceramics slightly increased at the sintering temperature of 960–980 °C. As SBTV1 ceramics sintered at 980 °C, several anisotropic elongate plate-like grains found, and the aspect ratio of the anisotropic elongate plate-like grains were 4.6–4.9. At higher sintering temperatures (e.g., 1000–1040 °C), the SBTV1 ceramics exhibited anisotropic plate-like grains, the average grain size of the SBTV1 ceramics increased, and the aspect ratio of the disk-like grains increased to 9.4. These differences in the microstructure of the SBTV1 ceramics were primarily owing to the addition of V_2_O_5_, because the V_2_O_5_ acts as a liquid-phase sintering aid and facilitate the growth of the (00*l*) planes^16^. The high density of the SBT ceramics were obtained as the sintering temperature set to 1280 °C for 3 h^11^. By comparing the results presented in Fig. 6 and Ref.^11^, it is evident that the addition of V_2_O_5_ in SBT ceramics can reduce the sintering temperatures and enhance the densify of the SBT-based ceramics.

**Figure 7:**
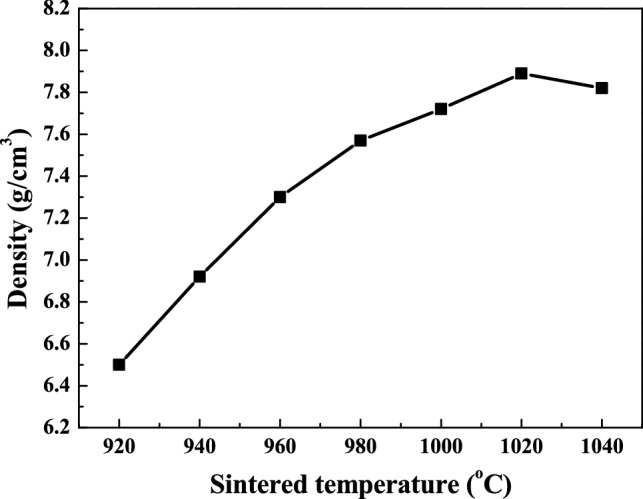
Density of SrBi_2_Ta_1.9_V_0.1_O_9_ ceramics under different sintering temperatures.

The densification procedures of the SBTV1 ceramics, sintered at different temperatures, are shown in Fig. 7. As the Fig. 7 shows, the bulk densities of the SBTV1 ceramics critically increase as the sintering temperatures increase from 920 to 1040 °C and reach the maximum values at 1020 °C. Either higher or lower sintering temperatures may cause the bulk densities of the SBTV ceramics to decrease. As sintering temperatures were lower than 1020 °C, the decrease of the porosity may cause this result. The porosity of the SBTV1 ceramics increased as the sintering temperatures were higher than 1020 °C. Following two reasons cause these results: (i) The upright plate-like and horizontal plate-like grains were observed on the SBTV1 ceramic surface, this phenomenon leading to the pores and decrease the bulk density. (ii) The large disk-typed grains grow at the expense of small ones, which result in the formation of new and larger voids where the small disk-typed grains were originally located. As the large disk-typed grains come into contact, continual growth pushes them away from one another, which causes expansion of the sintered compacts and result in decrease in bulk density.

**Figure 8:**
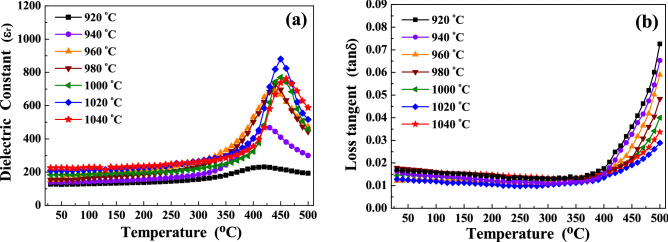
Temperature-dependence of (**a**) dielectric constants and (**b**) loss tangent of SrBi_2_Ta_1.9_V_0.1_O_9_ ceramic as a function of sintering temperature.

In Fig. S2, the dielectric constant (ε_r_) of the SBTV1 ceramics as a function of the measurement temperature and frequency. The dielectric constants of the SBTV1 ceramics increased with increasing measurement temperature and reached their maximum at the transition temperature, as shown in Fig. S2a–g. Then, the dielectric constant of the SBTV1 ceramics decreased as future increased measurement temperature, except for measurement at 10 kHz. Figure 8 presents the temperature dependence of the dielectric constant (ε_r_ − T) and loss tangent (tan δ − T) curves of the SBTV1 ceramics at 1 MHz. As observed in Fig. 8a, the maximum dielectric constant (the dielectric constant at Curie temperature) of the SBTV1 ceramics first increased from ε_r(max)_ = 234 to ε_r(max)_ = 887 as the sintering temperature was increased from 920 to 1020 °C; it then slightly decreased (ε_r(max)_ = 762) when the sintering temperature at 1040 °C. Compared with SBT ceramics, the SBTV1 ceramics had a higher maximum dielectric constant and a lower sintering temperature of approximately 1020 °C^19^. Even at 940 °C, the maximum dielectric constant was approximately 483, higher than that of SBT and SBT-base ceramics^19–21^. Hence, the crystallization and grain size of the SBTV1 ceramics is more favorable than those of SBT ceramics. Thus, in addition to the effect of the lower melting temperature of V_2_O_5_, the better crystallization and grain size of the SBTV1 ceramic could be attributed to the liquid-phase sintering effect that was caused by the low-temperature eutectic bonding in the SrO–V_2_O_5_ system^22^. From the XRD patterns presented in Fig. 1, we infer that the dielectric constant of SBTV1 ceramics is higher than that of SBT ceramics and that this is due to the increase in the preferred orientation of the (00*l*) planes of the SBTV1 ceramics. The increase in the preferred orientation of the (00*l*) planes increases the polarizability, which in turn increases the dielectric constant. At a sintering temperature of 1040 °C, the maximum dielectric constant of the SBTV1 ceramics decreased to 762. This result may be due to the following two reasons. First, the higher sintering temperature decreases the density owing to the evaporation of the low-melting-point V_2_O_5_ phase (shown in Fig. 7)^14^. Second, as presented in Fig. 6f, some upright plate-like grains were observed at the surface, and pores, which have low a dielectric constant, increased evidently. Figure 8b presents the tangent loss (tan δ) of the SBTV1 ceramics as a function of sintering temperature. The addition of V_2_O_5_ in the SBT ceramics significantly reduced the dielectric loss of SBT-based ceramics. When Ta^+5^ ions are substituted with V^+5^ ions in the SBT ceramics, the degree of densification of the SBTV1 ceramic increased as the sintering temperature increases from 920 to 1020 °C, and in turn significantly decreases the dielectric loss.

**Figure 9:**
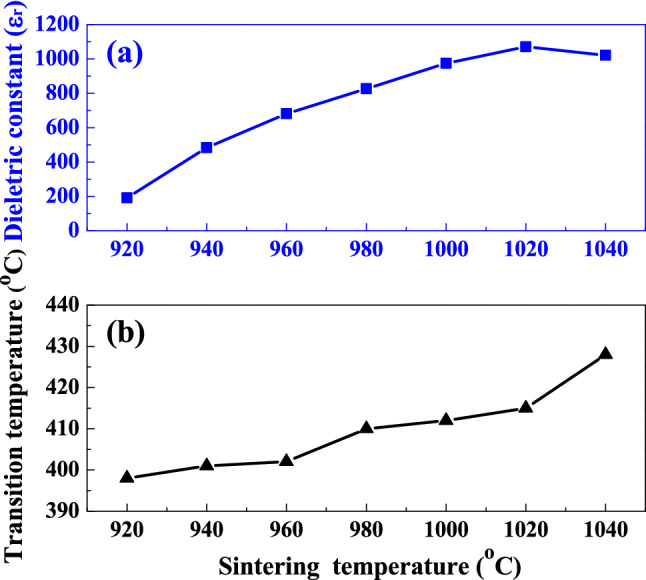
(**a**) Maximum dielectric constant and (**b**) Curie temperature of SrBi_2_Ta_1.9_V_0.1_O_9_ ceramic as a function of sintering temperature.

Figure 9 shows the maximum dielectric constant and Curie temperature of SBTV1 ceramic as a function of sintering temperature. The Curie temperature (the temperature that yields the maximum dielectric constant) of the SBTV1 ceramics was higher than those of SBT ceramics reported in the literature^19,20^. The Curie temperature of the SBTV1 ceramics increased from 389 to 438 °C as the sintering temperature increased from 920 to 1040 °C (Fig. 9b). It know that the higher was the amount of V_2_O_5_ used as a substitute for Ta_2_O_5_ in the SBT ceramics, the higher was the Curie temperature, suggesting that at higher sintering temperatures, a higher amount of V_2_O_5_ is substituted at the Ta_2_O_5_ site in SBT ceramics (shown in Fig. 9b), because of which the Curie temperature increases. The high Curie temperature is also indicative of enhanced polarizability^14,22^, which verifies the aforementioned reason for the increase in the dielectric constant of SBTV1 ceramics.

The polarizability of isotropic perovskite ferroelectrics with the ABO_3_ structure is strongly influenced by the size of the *A* and *B* site cations. As the *A* site cation is substituted with a larger ion, the size of the oxygen ion at the octahedral corner increases, thus increasing the polarizability and transition temperature of the material. As the *B* site cation inside the oxygen octahedra is replaced by a smaller ion, the “rattling space” available for the *B* site ion increases. Thus, polarizability increases and so does the Curie temperature. These results indicate that the increase in the transition temperature is due to the substitution of smaller V^5+^ ions (V^5+^ = 0*.*54 Å) at the Ta^5+^ ion site (Ta^5+^  = 0*.*64 Å). Similar results have been reported for the SBN-added V system when partial pentavalent Nb^+5^ ions were substituted by pentavalent V^5+^ ions^23^.

**Figure 10:**
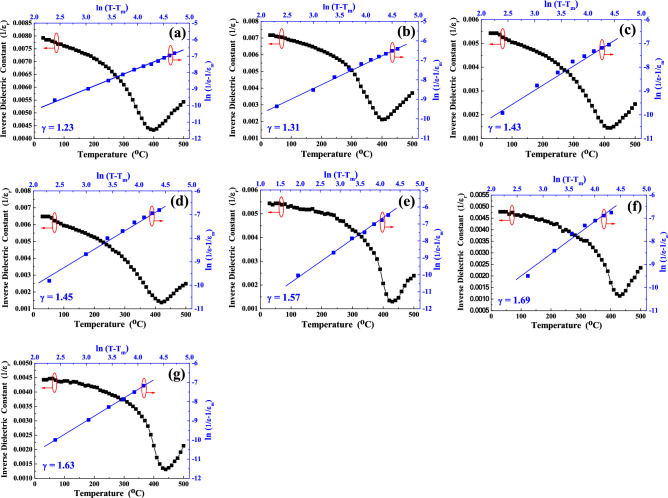
Plots for the temperature − 1/ε and log(1/ε − 1/ε_m_) − log(T − T_m_) curves of SrBi_2_Ta_1.9_V_0.1_O_9_ ceramics with different sintered temperature. (**a**) 920 °C, (**b**) 940 °C, (**c**) 960 °C, (**d**) 980 °C, (**e**) 1000 °C, (**f**) 1020 °C and (**g**) 1040 °C, respectively.[Symbols: experimental data; Solid line: simulation data].

In order to find SBTV1 ceramics with the properties of the normal-typed or relaxor-typed ferroelectric structures, the Curie–Weiss law and the modified Curie–Weiss law are used to analyze the ε_r_–T curves. Figure 10a–g shows the plot of inverse dielectric constant versus temperatures of SBTV ceramics with different sintered temperature. We know that the dielectric constant of a normal-type ferroelectric above the Curie temperature follows the Curie–Weiss law described by^24^

6 $${ }\varepsilon^{\prime} = \frac{C}{{T - T_{0} }}$$

where T_0_ is the Curie–Weiss temperature, C is the Curie–Weiss constant, T is the measured temperature and T > T_C_. Degree of deviation from the Curie–Weiss law can be defined by ΔT_m_ as the following:

7 $$\Delta T_{m} = T_{cw} - T_{m}$$

where T_cw_ denotes the temperature from the dielectric constant starts to deviate from the Curie–Weiss law, and T_m_ represents the temperature having the maximum dielectric constant. The T_C_ is determined from the graph by extrapolation of the reciprocal of dielectric constant of the paraelectric region and the values obtained are given in Fig. 10. In the Fig. 10, three different regions may be distinguished: the ferroelectric state at temperatures below the T_m_, the state where polar clusters exist for temperatures between T_m_ and T_cw_, and the paraelectric state for temperatures above T_cw_. A modified Curie–Weiss law has been proposed by many research groups to account of the diffuseness of a phase transition as^24^

8 $$\frac{1}{{\varepsilon^{{{\prime}}} }} - \frac{1}{{\varepsilon_{{\text{m}}} }} = \frac{{\left( {T - {\text{T}}_{{\text{m}}} } \right)^{\gamma } }}{{{\text{C}}^{\prime} }}$$

where γ and C′ are assumed to be constants, the γ value is between 1 and 2 and it gives information on the characteristic of the phase transition. γ = 1 is the case for normal-typed ferroelectrics and γ = 2 is caused for the ideal relaxor-typed ferroelectrics characteristics^25,26^. The plots of log(1/ε′–1/ε_m_) vs. log(T–T_m_) for SBTV1 ceramics measured at 1 MHz are shown in Fig. 10, the linear relationship was observed in SBTV1 ceramics, and the slopes of the fitting curves were used to determine the γ value. After fitting the experimental data to the modified Curie–Weiss law, it obtain the value of parameter γ = 1.23, 1.31, 1.43, 1.45, 1.57, 1.69 and 1.63, respectively for sintered temperature increased from 920 to 1040 °C. It found that the most part of the SBTV1 ceramics shows the normal-typed ferroelectrics characteristics. As the sintered temperature at 1000 °C to 1040 °C (Fig. 10e–g), the SBTV1 ceramics reveal the strong relaxor-typed ferroelectrics characteristics than the normal-typed ferroelectrics characteristics. When the SBTV1 ceramics sintered in the air environment, the oxygen vacancies could be generated and owing to the existence of the reduced valence state of V^5+^ ions. As found in the SBTV1 system, although it is planned to substitute pentavalent Ta^5+^ ions, with pentavalent V^5+^ ions, the tetravalent V^4+^ ions may form and enter the B sites of the layered perovskite structure. In order to keep the electroneutrality, one oxygen vacancy and two V^4+^ ions entering the crystal structure, and the entire reaction is description by the following equation:

9 $$2V^{5 + } + O_{O}^{X} \to 2V^{4 + } + V_{O}^{ \cdot \cdot } + \frac{1}{2}O_{2} \left( g \right)$$

where $V_{O}^{ \cdot \cdot }$ is the oxygen vacancy with two effective positive charges and $O_{O}^{X}$ is the oxygen ion in oxygen site. At high temperatures, the contribution degree of the dielectric relaxation caused by the oxygen vacancy is larger than that at low temperatures. The dielectric relaxation caused by the oxygen vacancy is a diffusion-related process, which is an exponentially dependent on temperature. At lower temperatures (920 °C to 980 °C), there is not enough energy to overcome the diffusion energy barrier and the oxygen vacancy induced dielectric relaxation is negligible. At high temperatures (1000 °C to 1040 °C), there is enough energy and the contribution of the oxygen vacancy induced dielectric relaxation becomes significant.

**Figure 11:**
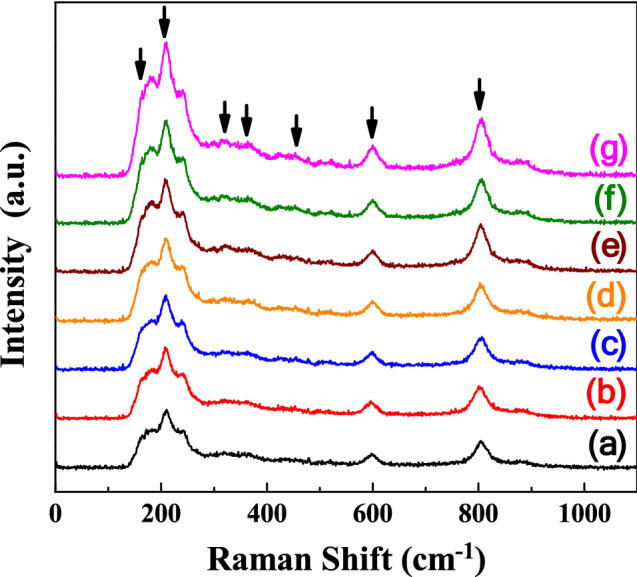
Raman spectra of SrBi_2_Ta_1.9_V_0.1_O_9_ ceramic with different sintered temperatures. (**a**) 920 °C, (**b**) 940 °C, (**c**) 960 °C, (**d**) 980 °C, (**e**) 1000 °C, (**f**) 1020 °C, and (**g**) 1040 °C, respectively.

**Figure 12:**
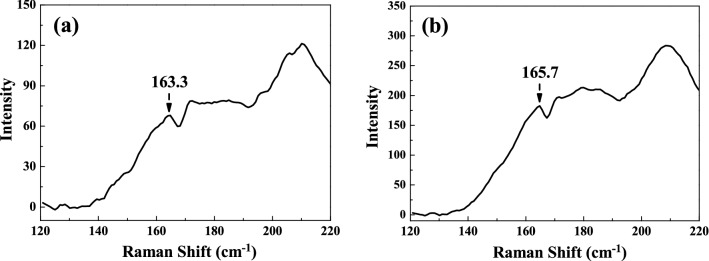
Raman spectra of SrBi_2_Ta_1.9_V_0.1_O_9_ ceramic at 163 cm^−1^ band with different sintered temperature. (**a**) 920 °C and (**b**) 1040 °C.

The sintering temperature–dependent Raman spectra of the SBTV1 ceramics are presented in Fig. 11. Due to instrument limitations, Raman bands at frequencies lower than 100 cm^−1^ could not be observed. Other studies have reported that the Raman spectrum of a SBT ceramics exhibit intense peaks at approximately 163, 210, 600, and 805 cm^−1^ and that the other peaks at 319, 356, and 455 cm^−1^ corresponded to weak features^27–29^. Our result was in agreement with these characteristic bands as bands at approximately 163, 210, 319, 356, 455, 600, and 805 cm^−1^ were prominent in the SBTV1 ceramics in this study. The band at 163.3 cm^−1^ is associated with the lattice vibration of the Ta^+5^ ions along the *z* direction (TO mode A_1g_)^30^. At higher sintering temperatures, the band at 163.3 cm^−1^ shifted upward to 165.7 cm^−1^ (Fig. 12a,b). The nondegenerate A_1g_ mode, vibrate in the plane perpendicular to the c axis, and the shift occurs because the Ta^+5^ ions are replaced by the lower mass V^+5^ ions. The A_1g_ mode shifts to higher/lower wave number is relation to the grain growth, therefore, the grain size [faster growth of the (00*l*) planes] of the SBTV1 ceramic increases, and it will be leading to the A_1g_ mode shifts to higher wave numbers, as shown in Figs. 1 and 6. In addition, it known that the A_1g_ mode behavior is responsible for the phase transition in SBTV1 sample. This result is in line with the variation in the Curie temperature presented in Fig. 9b. The TO mode of SrO with a rock salt structure revealed at the band at 210 cm^−1^^31,32^. The peaks at approximately 600 and 805 cm^−1^ are attributed to the internal vibration of the TaO_6_ octahedron. However, the oxygen ions contributing to the two bands are different. The band at 600 cm^−1^ can be attributed to the vibration of the oxygen ion (O_2_) at the apex of the TaO_6_ octahedron. Moreover, the band at 805 cm^−1^ can be attributed to the vibration of oxygen ions (O_4_, O_5_) in the TaO_6_ octahedron.

**Figure 13:**
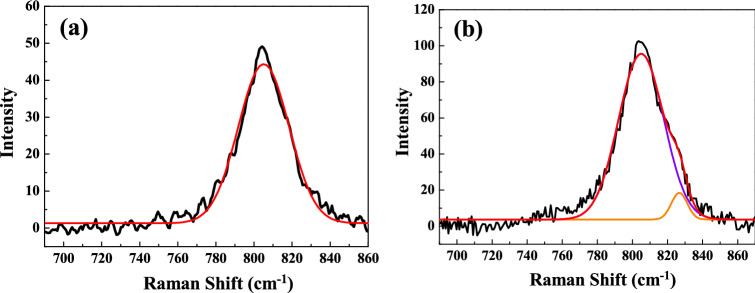
De-convolution Raman spectra of SrBi_2_Ta_1.9_V_0.1_O_9_ ceramic at 810 cm^−1^ band with different sintered temperature. (**a**) 920 °C and (**b**) 1040 °C.

For better understanding about this mode, the de-convolution of SBTV1 ceramic Raman spectra at 805 cm^−1^ band by using the Gaussian fit is shown in Fig. 13. It is found that the single peak at 805 cm^−1^ shown in Fig. 13a was corresponding to TaO_6_ mode. As sintering temperature was 1040 °C, the peaks at 805 cm^−1^ and 826.7 cm^−1^ were corresponding to TaO_6_ mode and B-site substitution modes, respectively^32^. The peak’s intensity of 826.7 cm^−1^ increased with sintering temperature. Therefore, the TaO_6_ and VO_6_ octahedral structures exist in SBT ceramic as V_2_O_5_ is added. The substitution of V_2_O_5_ on Ta_2_O_5_ sites of SBT ceramic apparently changes the high frequency Raman spectra of SBTV1 ceramic. Because the V_2_O_5_ is pentavalent element and it is substituted inside the octahedral cage, the lowest Raman modes of SBTV1 ceramic do not show any appreciable variation, only the 163.3 cm^−1^ band is shifted to 165.7 cm^−1^.

**Figure 14:**
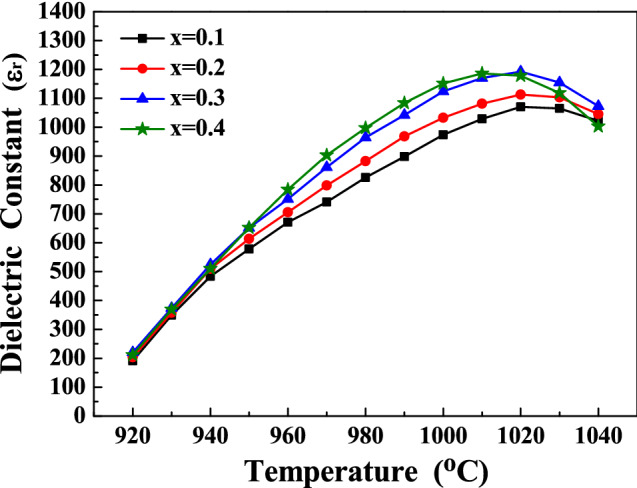
Temperature-dielectric constant curves of the SrBi_2_(Ta_2-x_V_x_)O_9_ ceramics as a function of V_2_O_5_ contents. (**a**) x = 0.1, (**b**) x = 0.2, (**c**) x = 0.3, (**d**) x = 0.4.

In order to understand the V_2_O_5_ content effect of the SBTV ceramics, the SBTV1, SrBi_2_(Ta_0.8_V_0.2_)O_9_ (SBTV2), SrBi_2_(Ta_0.7_V_0.3_)O_9_ (SBTV3) and SrBi_2_(Ta_0.6_V_0.4_)O_9_ (SBTV4) ceramics sintered at 920 to 1040 °C were further investigated. Figure 14 shows the dielectric constants of the SBTV1, SBTV2, SBTV3 and SBTV4 ceramics as a function of sintering temperature. The dielectric constant of the SBTV1 ceramics increased as the sintered temperature increased, and the maximum dielectric constant obtained at 1020 °C sintering temperature. The same phenomenon were find in the SBTV2, SBTV3 and SBTV4 ceramics. As the V_2_O_5_ content increases from x = 1 to x = 3, the dielectric constant of the SBTV ceramic sintered at 1020 °C increases from ε_r_ = 1070.6 to ε_r_ = 1192.3, than the dielectric constant decreases as the V_2_O_5_ content future increases at x = 0.4. A subtle crystallographic evolution of the SBTV ceramics occurred along with the increasing V_2_O_5_ doping content. Based on the surface image of the SBTV1 ceramics presented in Fig. 12a, several anisotropic plate-like grains were observed and the average aspect ratio of the anisotropic plate-like grains was 7.17. The average aspect ratio of the anisotropic plate-like grains of the SBTV2, SBTV3 and SBTV4 ceramics are 8.55, 8.76 and 8.18, respectively, as shown in Fig. 12b–d. The different aspect ratios of the SBTV1, SBTV2, SBTV3 and SBTV4 ceramics were caused by the addition V_2_O_5_ and promotes the growth of the (00*l*) planes.

**Figure 15:**
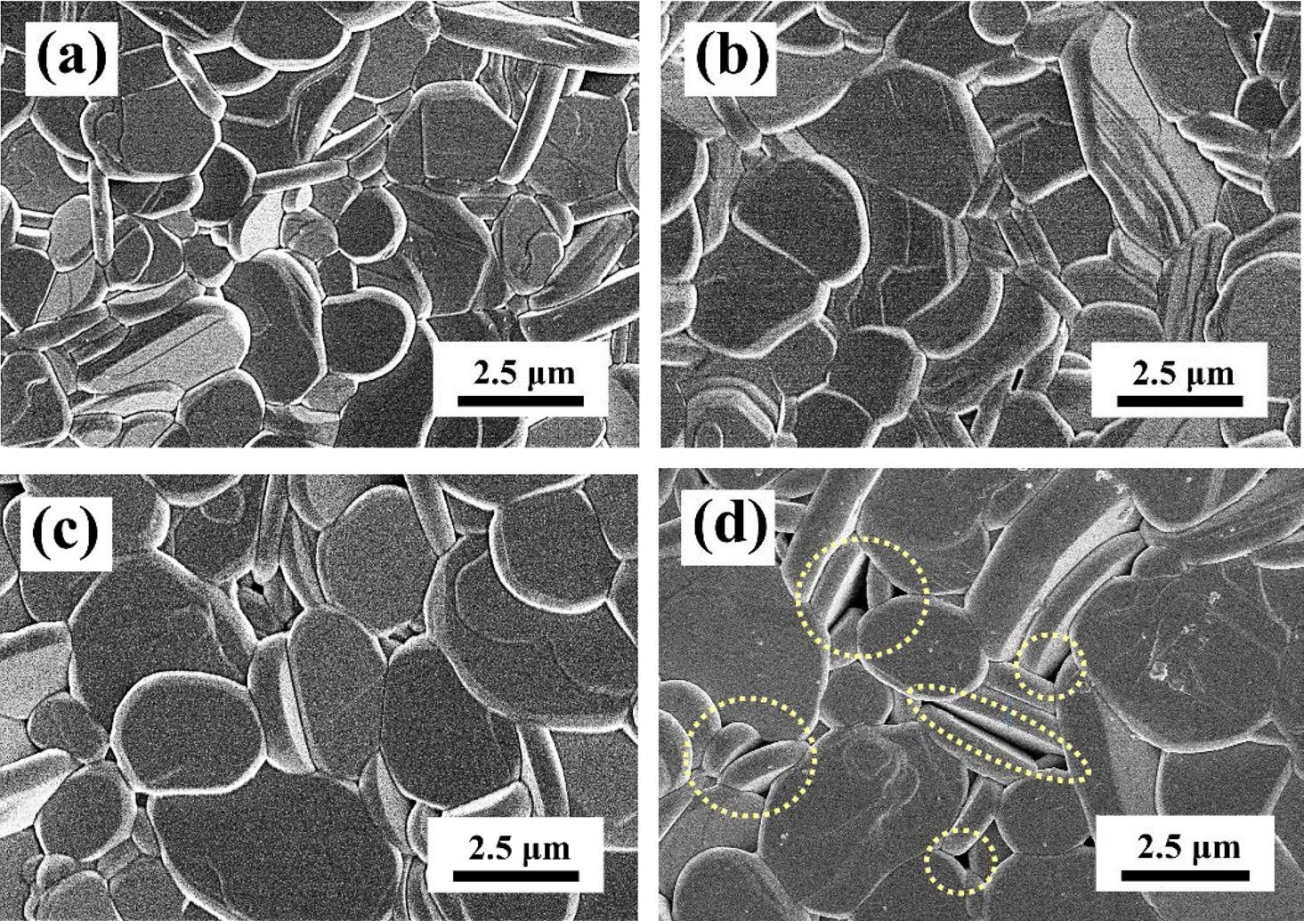
SEM images of the SrBi_2_ (Ta_2−x_V_x_)O_9_ ceramics as a function of V_2_O_5_ contents. (**a**) x = 0.1, (**b**) x = 0.2, (**c**) x = 0.3, (**d**) x = 0.4.

However, the small pores on the SBTV4 ceramics were more obvious compared with those on the SBTV2, SBTV3 and SBTV4 ceramics presented in Fig. 15a–c. Consequently, the dielectric constant of the SBTV4 ceramics ceramic sintered at 1020 °C is small than the SBTV3 ceramics. This result is caused by the upright anisotropic plate-type grains that grow on the plane anisotropic plate-type of the SBTV4 ceramic. The sintered temperature of the SBTV4 ceramic is higher than the ideal temperature, it lead to the grain growth rate is too fast, not only (00*l*) planes but other planes. Therefore, the average aspect ratio of the anisotropic plate-like grains of the SBTV4 ceramic is small than the SBTV3 ceramic.

**Figure 16:**
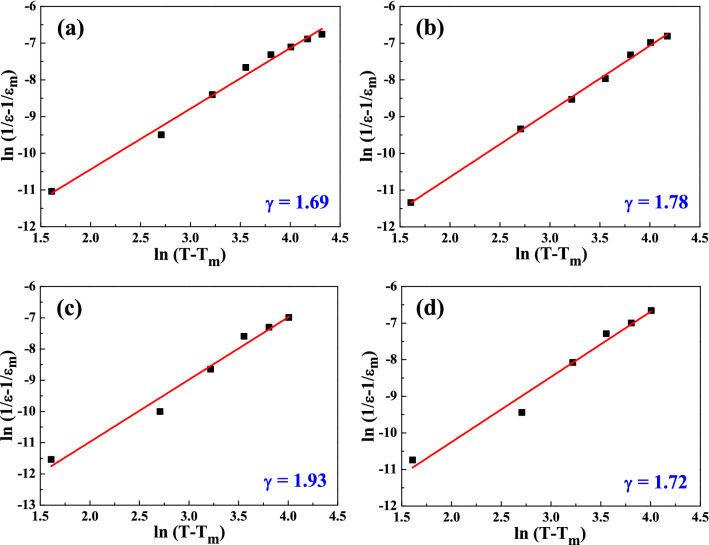
Plots for the temperature − 1/ε and log(1/ε − 1/ε_m_) − log(T − T_m_) curves of SrBi_2_(Ta_2−x_V_x_)O_9_ ceramics with different sintered temperature. (**a**) x = 0.1, (**b**) x = 0.2, (**c**) x = 0.3, (**d**) x = 0.4.[Symbols: experimental data; Solid line: simulation data].

The plot of log(1/ε′ − 1/ε_m_)—log(T − T_m_) for SrBi_2_(Ta_2−x_V_x_)O_9_ ceramics with x = 0.1 to x = 0.4 measured at 1 MHz is represented in Fig. 16. A linear relationship is observed in the SBTV3 ceramics, and the γ value are determined by the slopes of the fitting curves. After fitting the experimental data by the modified Curie–Weiss law, the γ values of SBTV3 ceramics are 1.69, 1.78, 1.93, 1.93, and 1.72 as x = 0.1, x = 0.2, x = 0.3, x = 0.4, respectively. The γ values of the SBTV1, SBTV2, SBTV3, SBTV4 ceramics were compared. The results demonstrated that all the SrBi_2_(Ta_2−x_V_x_)O_9_ ceramics with different V_2_O_5_ content sintered at 1020 °C present a relaxor-type ferroelectric characteristics and the maximum γ value obtained at the SBTV3 ceramic. Relaxor-type that are characterized by diffuse phase transitions, are of significant interest for various applications as these possess exceptionally high dielectric and high Curie temperatures responses over a wide range of temperatures relatively.

## Conclusions

Aurivillius family of oxides have become increasingly important as lead-free ferroelectric materials. Though the majority of Aurivillius compounds have normal ferroelectric to paraelectric phase transitions, a few compounds however exhibit relaxor-type properties. In this study, The Ta_2_O_5_ replaced by the V_2_O_5_ in SrBi_2_(Ta_2−x_V_x_)O_9_ (SBTV) ceramics, the sintering temperature was reduced, the densification was enhanced, and the dielectric constant of SrBi_2_(Ta_2−x_V_x_)O_9_ ceramics was improved. As the sintering temperature increased, from 980 to 1040 °C, the degree of the c-axis preferential orientation for the SrBi_2_(Ta_1.9_V_0.1_)O_9_ ceramics increased, and the c-axis preferential orientation for the SrBi_2_(Ta_2−x_V_x_)O_9_ ceramics slightly increased as the V_2_O_5_ contents was increased from x = 0.1 to x = 0.4, and disk-like grains were observed in the ceramics. Compared with strontium bismuth tantalite (SBT) ceramics, significantly higher maximum dielectric constants and significantly higher Curie temperatures were obtained for SrBi_2_(Ta_2−x_V_x_)O_9_ ceramics. Raman spectroscopy was used to study the lattice vibrational modes and structural transition of the SrBi_2_(Ta_2−x_V_x_)O_9_ ceramics. The high-frequency Raman spectrum evidenced the presence of octahedral TaO_6_ and VO_6_ when V_2_O_5_ was incorporated in the ceramics. The V^+5^ ions that occupied the B sites lowered the sintering temperature and improved the crystallization plane, dielectric properties, and Curie temperature of the SBT ceramics. The SrBi_2_(Ta_1.9_V_0.1_)O_9_ ceramics revealed that the normal-type ferroelectric characteristic as the sintered temperature were 920 °C, 940 °C, 960 °C and 980 °C, respectively. Then the relaxor-type ferroelectric characteristics of the SrBi_2_(Ta_2−x_V_x_)O_9_ ceramics sintered at 1020 °C were obtained when V_2_O_5_ contents was increased from x = 0.1 to x = 0.4. Relaxor that are characterized by high dielectric of wide range of temperatures and high Curie temperatures, and it make them attractive for nonvolatile memory applications.

## Supplementary information

Supplementary Information.

## Data Availability

The datasets supporting the conclusions of this work are included within the article. Any raw data generated and/or analyzed in the present study are available from corresponding authors on request.

## Abbreviations

SBTV: Strontium bismuth tantalate vanadate
SBT: Strontium bismuth tantalite
BLSF: Bismuth layer-structured ferroelectric
PZT: Lead zirconate titanate
RoHS: Restriction of hazardous substances directive
SEM: Scanning electron microscopy
XRD: X-ray diffraction
FWHM: Full width at half maximum
SBTV: SrBi_2_(Ta_2−x_V_x_)O_9_
SBTV1: SrBi_2_(Ta_1.9_V_0.1_)O_9_
SBTV2: SrBi_2_(Ta_1.8_V_0.2_)O_9_
SBTV3: SrBi_2_(Ta_1.7_V_0.3_)O_9_
SBTV4: SrBi_2_(Ta_1.6_V_0.4_)O_9_
