# Publisher Correction: Effect of V_2_O_5_ B-site substitution on the microstructure, Raman spectrum, and dielectric properties of SrBi_2_Ta_2_O_9_ ceramics

**DOI:** 10.1038/s41598-021-85084-x

**Published:** 2021-03-09

**Authors:** Chia‑Ching Wu, Cheng‑Fu Yang

**Affiliations:** 1 Department of Applied Science, National Taitung University, Taitung, Taiwan, R.O.C.; 2 Department of Chemical and Materials Engineering, National University of Kaohsiung, Kaohsiung, Taiwan, R.O.C.

Correction to: *Scientific Reports* 10.1038/s41598-020-73327-2, published online 05 November 2020

In the original version of this Article, Cheng-Fu Yang was omitted as a corresponding author. Correspondence and request for materials should be addressed to Chia-Ching Wu and Cheng-Fu Yang. This error has now been corrected in the HTML and PDF versions of the Article.

