# Supplementary material for: Effect of V2O5 B-site substitution on the microstructure, Raman spectrum, and dielectric properties of SrBi2Ta2O9 ceramics
Source: Sci Rep. 2020 Nov 5;10:19147. doi: 10.1038/s41598-020-73327-2 (PMC7644718; doi:10.1038/s41598-020-73327-2)
Supplement: Supplementary file 1 — Supplementary Information. [file 41598_2020_73327_MOESM1_ESM.docx]

**Supplementary information**

**Effect of V_2_O_5_ B-site Substitution on the Microstructure, Raman Spectrum, and Dielectric Properties of SrBi_2_Ta_2_O_9_ Ceramics**

**Chia-Ching Wu^1,*^ and Cheng-Fu Yang^2,*^**

^1^ Department of Applied Science, National Taitung University, Taitung, Taiwan, R.O.C.

Corresponding author. Email: [ccwu@nttu.edu.tw](mailto:ccwu@nttu.edu.tw)

^2^Department of Chemical and Materials Engineering, National University of Kaohsiung, Kaohsiung, Taiwan, R.O.C.

Corresponding author. Email: [cfyang@nuk.edu.tw](mailto:cfyang@nuk.edu.tw)

Figure S1 Rietveld refinement performed for the SrBi_2_Ta_1.9_V_0.1_O_9_ ceramics sintered at different temperatures. (a) 920 ^o^C, (b) 960 ^o^C, (c) 1000 ^o^C, and (d) 1040 ^o^C. (SrBi_2_Ta_2_O_9_ Structural parameters: a=5.5212 Å, b=5.5215 Å, c=24.992 Å)

Figure S2 Dielectric constant of the SrBi_2_Ta_1.9_V_0.1_O_9_ ceramics measured at different frequency. (a) 920 ^o^C, (b) 940 ^o^C, (c) 960 ^o^C, (d) 980 ^o^C , (e) 1000 ^o^C , (f) 1020 ^o^C and (g) 1040 ^o^C.
